# Use of a Non-parametric Bayesian Method to Model Health State Preferences: An Application to Polish and Irish EQ-5D-5L Valuations

**DOI:** 10.3389/fpubh.2022.917728

**Published:** 2022-06-23

**Authors:** Samer A. Kharroubi, Dan Kelleher

**Affiliations:** ^1^Department of Nutrition and Food Sciences, Faculty of Agricultural and Food Sciences, American University of Beirut, Beirut, Lebanon; ^2^School of Health and Related Research, The University of Sheffield, Regent Court, Sheffield, United Kingdom; ^3^Yale School of Public Health, Yale University, New Haven, CT, United States

**Keywords:** non-parametric Bayesian methods, preference-based health measures, EQ-5D-5L, composite time trade-off, health-related quality of life

## Abstract

Valuations of preference-based measures for health are conducted in different countries. There is scope to use results from existing countries' valuations to generate better valuation estimates than analyzing the data from each country separately. We analyse data from two smaller design EQ-5D-5L valuation studies where a sample of 119 Polish migrants and 123 native Irish valued 30 common health states using similar composite time trade-off protocols. We apply a non-parametric Bayesian method to provide better predictions of the Polish (Irish) population utility function when the existing Irish (Polish) results were used as informative priors. The resultant new estimates were then compared to those obtained by analyzing the data from each country by itself *via* different prediction criterions. The results suggest that existing countries' valuations could be used as potential informative priors to produce better valuation estimates under all prediction criterions used. The implications of these results will be hugely important in countries where valuation studies are expensive and hard to conduct. Future application to other countries and to other preference-based health measures are encouraged.

## Introduction

Several preference-based measures of health-related quality of life (HRQoL) are currently available. Such generic measures include the EuroQol five-dimensional (EQ-5D) questionnaire (1), health utilities index 2 (HUI2) and 3 (2, 3), assessment of quality of life (4), Quality of Well-being scale (QWB) (5), and the six-dimensional health state short form (SF-6D) (6) along with disease-specific measures (7, 8). All of these measures produce derived health-state utilities that can be used for computing quality-adjusted life-years (QALYs) for use in cost-effectiveness analyses (9).

The EQ-5D is the most commonly used preference-based measure of HRQoL. It describes five multi-level dimensions of health: mobility, self-care, usual activities, pain/discomfort and anxiety/depression. Two versions of the instrument are available: the three level EQ-5D (EQ-5D-3L) allowing the determination of 3^5^ = 243 health states and the five level EQ-5D (EQ-5D-5L) allowing the determination of 5^5^ = 3,125 health states (10, 11). The EQ-5D-5L, though, improves the sensitivity and discriminatory potential of the EQ-5D (12, 13). The EQ-5D-5L has been valued in more countries than any of the other generic measures, thereby there are many different value sets from different countries and subgroups available (14–22).

In the context when plenty of data on each country is available, good utility estimates for each country can be produced by analyzing its data separately. However, in the case when limited quantity of data on some (or all) countries is available, it is argued that combined analysis may generate better estimation of every country's utility estimates than analyzing its data by itself. This sort of analysis (adopting strength from existing countries) will be greatly important in countries where large-scale evaluation exercises are very expensive and hard to conduct, especially for countries with smaller populations or low- and middle-income countries (LMIC).

Advancement in statistical modeling techniques, such as Bayesian inference methods (23), offers the potential for borrowing strength from existing countries. In particular, it offers the potential for using the existing results of country 1 to improve those in country 2 by using the results in country 1 as informative priors. As such, the resultant utility estimates of country 2 could be more precise than modeling its own data separately. A number of researchers have investigated the use of such approach. For example, Chan et al. (24) found that EQ-5D-3L health state utilities obtained from shrinkage estimation allow valuation studies with very low sample size to adopt strength from another valuation studies to help improve precision in the estimated mean health utilities and reduce uncertainty. Kharroubi (25) developed a non-parametric Bayesian method that allows the already existing results from one country to be employed as a potential prior information in another country, and applied this method for analyzing the US EQ-5D-3L valuation dataset alongside the available UK dataset (26). In (27–32), Kharroubi et al. extended this work further to handle the SF-6D Hong Kong, Japan and Lebanon alongside the already available UK dataset, respectively.

Our primary purpose in the present paper is to investigate the use of aforementioned method for countries with smaller design valuation studies and different population compositions, type of work, cultures and languages, all of which could have an impact, suggesting that analyzing its own data separately may not always generate precise valuation estimates. This is investigated using a case study for Polish migrants and native Irish data modeling Irish (Polish) data alongside small Polish (Irish) samples to generate Polish (Irish) estimates. Despite the present paper not offering new methodological advances as the model presented here is a replication of that already reported in Kharroubi et al. (25–32) papers, it further tells a reassuring story regarding the superiority and flexibility of the non-parametric Bayesian approach in using existing preference data, thereby generating accurate estimates.

The Polish and Irish EQ-5D-5L valuation surveys and study methods alongside the corresponding datasets are summarized in Section 2. In the next section, the Bayesian non-parametric model is described whereas the results are reported in Section 3. In the last section, the results are discussed and some limitations and suggestions for further research are set out.

## Materials and Methods

### The EQ-5D-5L

The EQ-5D-5L describes five multi-level dimensions of health: mobility, self-care, usual activities, pain/discomfort and anxiety/depression. Each dimension is assigned to five levels of health-related problems: no problems “1,” slight problems “2,” moderate problems “3,” severe problems “4,” and extreme problems “5” (11). Different combinations allow the determination of 3,125 distinct health states, each of which is associated with a five-digit identifier, beginning from 11,111 for best health state (perfect health) and ending with 55,555 for the worst health state, referred to as “the pits.”

### Survey Design and Sampling Strategy

Data from two smaller design EQ-5D-5L valuation studies where, using similar composite time trade-off protocols (cTTO), valuations for 30 common health states were elicited from Polish migrants and native Irish, both living in Ireland. Since they represent the largest non-Irish community residing in Ireland, the Polish migrants were chosen to be included in this valuation study. Detailed description of the survey design and sampling strategy has been reported elsewhere (33). In brief, a sample of 240 (120 Polish migrants and 120 native Irish) respondents was recruited to value six practice states each in addition to one block of 11 cTTO states. The valuation study was conducted between June 2018 and September 2019 and the orthogonal design of the health states was provided by the EuroQol Research Foundation. All interview sessions were conducted using the EuroQol Portable Valuation Technology (EQ-PVT), which is a computer assisted personal interview software and protocol (34, 35). The valuation study has been ethically approved by the NUI Galway's Research Ethics Committee. Further details on the valuation study is provided elsewhere (33).

### Experimental Design

The orthogonal design was analogous to the one applied in Yang et al. (34). A sample of 30 EQ-5D-5L health states was chosen for valuation. The sample contained 25 health states, including the ‘pits' state. A further five mild health states were selected, resulting a total of 30 EQ-5D-5L health states for valuation. A key advantage of using an orthogonal design is that it allows for a small number of health states to be valued and a small sample size to be used in comparison to that of a full national valuation study, which includes 86 EQ-5D-5L health states and a minimum sample size of 1,000 respondents (17). A comparative study between estimates of a smaller design EQ-5D-5L valuation study and that of the full national valuation study has been conducted in Yang et al. (34, 36). Results revealed that the smaller design EQ-5D-5L valuation study performed well in comparison to the larger design and that no significant changes in prediction errors have been obtained when modeling EQ-5D-5L cTTO data.

### Blocking

Using the blocking algorithm that is readily available in the “AlgDesign” package in the software package R, the 30 EQ-5D-5L health states were split up into three blocks of 11 health states. The rationale for this is to make sure that within-block variance is maximized, thereby observations on the full health–dead scale are attained [see Kelleher et al. (33) for an overview].

### Interviewers and Respondents

The EQ-5D-5L cTTO data were extracted from Polish migrants and native Irish, both living whole-time in Ireland. Full details on the valuation study is provided in Kelleher et al. (33). Prior to the survey, respondents were asked to state their country of birth and whether they reside in Ireland whole-time. The interviews were conducted by a group of seven interviewers plus one study coordinator. Respondents were contacted through a Facebook page, by email through the study coordinator, or through friends and family using snowball sampling. Respondents were also asked to provide written consent to be included in the study.

More detailed explanation on the extended experimental design, survey design and sampling strategy is provided in Kelleher et al. (33).

### Modeling

Kharroubi (25) developed a non-parametric Bayesian model that allows the already existing results from one country to be employed as a potential prior information in another country. Here we make use of this model to investigate whether the use of Polish (Irish) alongside the existing Irish (Polish) dataset generates more accurate utility estimates than modeling the data from each country alone. These resultant estimates are then compared in terms of different prediction criterions, including predicted against actual mean utility estimates, mean predicted error and root mean square error (RMSE).

Following Kharroubi (25), the non-parametric Bayesian model is defined as


(1) $$\begin{array}{l} y_{ij}=1-\{\alpha_{j}\left(1-u\left({\text{x}}_{ij}\right)\right\}+\varepsilon_{ij} \end{array}$$

where for *i* = 1, 2, …, *I*_*j*_ and *j* = 1, 2, …, *J*, **x**_*ij*_ is the *i*th health state evaluated by respondent *j* and the dependent variable *y*_*ij*_ is the respondent *j*'s cTTO valuation for that health state, α_*j*_ is a random effect of respondent *j* and ε_*ij*_ is the usual random error.

Assume that *t*_*j*_ is a vector of covariates for respondent *j* e.g., age, gender, socio-economic status or level of education. Kharroubi (25) then used the following distributions:


(2) $$\begin{array}{l} \alpha_{j}\sim LN\left(t_{j}^{T}\gamma ,\tau^{2}\right)\text{ and }\varepsilon_{ij}\sim N\left(0,v^{2}\right), \end{array}$$

where γ is the vector of unknown coefficients and τ^2^ and *v*^2^ are further unknown variance parameters to be estimated. That is, the distribution of the respondent effect α_*j*_ is then independent log-normal, resulting in a skewness that is also typically observed in valuation data, and ε_*ij*_ are independent normally distributed errors,

Note that, because of the way that the respondent effects have been modeled in distribution (2), the utility function u(x) turns out to be the median utility of health state x. Given it is an unknown function, it becomes a random variable in the Bayesian model, which in turn needs a prior belief. Kharroubi (25) formally assigned a multivariate normal distribution for *u*(**x**) with mean


(3) $$\begin{array}{l} E\left(u\left(\text{x}\right)|\beta \right)=E\left(u_{0}\left(\text{x}\right)|\text{y}\right)+\gamma +\beta^{T}\text{x} \end{array}$$

and variance–covariance matrix


(4) $$\begin{array}{l} \text{cov}\left(u\left(\text{x}\right),u\left({\text{x}}^{\prime }\right)|\sigma^{2}\right)=\text{cov}\left(u_{0}\left(\text{x}\right),u_{0}\left({\text{x}}^{\prime }\right)|\text{y}\right)+\sigma^{2}c\left(\text{x},{\text{x}}^{\prime }\right) \end{array}$$

where *E* (*u*_0_(**x**)|**y**) represents the average utility value for state **x** and $cov\left(u_{0}\left(\text{x}\right),u_{0}\left({\text{x}}^{\prime }\right)|\text{y}\right)$ denotes the variance–covariance matrix between the two utility functions *u*_0_(**x**) and $u_{0}\left({\text{x}}^{\prime }\right)\neg$ for two distinct health states **x** and **x**′, both of which are computed directly from modeling the existing countries' data. More details on this are given in Kharroubi (25).

Given equations (3) and (4), it is noteworthy that **x** = (*x*_1_, *x*_2_, …, *x*_5_) denotes a vector comprising discrete levels on each EQ-5D-5L dimensions and γ, β, and σ^2^ are unknown parameters. Note also that the regression parameters γ and β represent, respectively, the intercept term and the slopes as each of the 5 dimensions (mobility, self-care, usual activities, pain/discomfort and anxiety/depression) increases, whereas the term *c* (**x**, **x**′), defined below, represents the correlation between the two utility functions *u* (**x**) and *u* (**x**′) for two distinct health states **x** and **x**′ in the new country's data. As for equation (3), the prior mean *E* (*u* (**x**)|β) represents a prior belief about the utility function that it is approximately linear and additive in the different dimensions. In addition, the actual utility function is allowed to vary around this mean in accordance with to its multivariate normal distribution, and so it takes any form at all. With regards to equation (4), the correlation *c*(**x**, **x**′) decreases when the distance between **x** and **x**′ gets bigger. Kharroubi (25) defined *c*(**x**, **x**′) as


(5) $$\begin{array}{l} c\left(\text{x},{\text{x}}^{\prime }\right)=\exp\left\{-\sum b_{d}{\left(x_{d}-{x^{\prime }}_{d}\right)}^{2}\right\} \end{array}$$

where for *d* = 1, 2, …, 5, *x*_*d*_ and ${x^{\prime }}_{d}$- represents, respectively, the levels of dimension *d* in **x** and **x**′. The term *b*_*d*_ denotes a roughness parameter which by definition controls how close the actual utility function to a linear form in dimension *d*. For more explanation of this specific point, see Kharroubi (25).

In order to complete the Bayesian model, we need to assign prior distributions for hyperparameters γ, τ^2^, *v*^2^, β and σ^2^. Vague priors are usually specified unless specific prior information is available. Formally, we assign


(6) $$\begin{array}{l} p\left(\gamma ,\tau^{2},v^{2},\beta ,\sigma^{2}\right)\propto \tau^{-2}v^{-2}-\sigma^{-1}. \end{array}$$

Note that a flat prior was specified for σ, hence *p(*σ^2^) ∝ σ^−1^ (37). Note also that no prior distributions were assigned to the roughness hyper-parameters *b*_*d*_s. It is noted in Kharroubi et al. (23) that inference about *b*_*d*_s in Gaussian models is generally problematic, thus it is recommended to give them fixed values. We shall discuss one method to demonstrate this in section results.

We now formulate the posterior distribution of interest. Letting $u\;=\;{\left(u_{1},\;u_{2},\ldots ,u_{n}\right)}^{T}$ be the vector of utilities for the health states in the sample. Equations (3) and (4) give rise for the prior distribution of **u**


$$\begin{array}{l} \text{u}|\beta ,\;\sigma^{2}\;\sim \;N\left({\text{u}}_{0}+H\beta ,{C_{0}+\sigma }^{2}A\right) \end{array}$$

where


$$\begin{array}{l} H^{T}=\left(\text{h}\left({\text{x}}_{1}\right),\text{h}\left({\text{x}}_{2}\right),\ldots ,\text{h}\left({\text{x}}_{n}\right)\right),\text{h}\left(\text{x}\right)={\left(1,\text{x}\right)}^{T} \end{array}$$

and


$$\begin{array}{l} A=\left(\begin{array}{ccc} 1 & \cdots & c\left({\text{x}}_{1},{\text{x}}_{\text{n}}\right)\\ \vdots & \ddots & \vdots \\ c\left({\text{x}}_{\text{n}},{\text{x}}_{1}\right) & \cdots & 1 \end{array}\right). \end{array}$$

Note that **u_0_** and *C*_0_ are obtained from modeling the existing countries' data. Now let $\alpha \;=\;{\left(\alpha_{1},\;\alpha_{2},\ldots ,\alpha_{J}\right)}^{T}$ be the vector of respondent effects, then the posterior distribution of **θ** **= (u, α,** γ, τ^2^, *v*^2^, β, σ^2^) is


$$\begin{array}{l} p\left(\theta \right)\propto {v^{2}}^{-\frac{I}{2}-1}\;\exp\{-\frac{1}{2v^{2}}\sum_{j=1}^{J}\sum_{i=1}^{I_{j}}(y_{ij}\\ \;-1-\alpha_{j}{\left(1-u\left({\text{x}}_{ij}\right)\right)}^{2}\}{\;}^{*}\\ \;|{C_{0}+\sigma }^{2}A{|}^{-\frac{1}{2}}\;\exp\{-{\left(\text{u}-{\text{u}}_{0}-H\beta \right)}^{T}{\left({C_{0}+\sigma }^{2}A\right)}^{-1}\\ \;{\left(\text{u}-{\text{u}}_{0}-H\beta \right)/2\}}^{*} \end{array}$$

(7) $$\begin{array}{l} {\left(\tau^{2}\right)}^{-\frac{J}{2}-1}\overset{J}{\underset{j=1}{\prod }}{\alpha_{j}}^{-1}\;exp\left\{-{\left(\ln\left(\alpha_{j}-{\text{t}}_{j}^{T}\gamma \right)\right)}^{2}/\left(2\tau^{2}\right)\right\} \end{array}$$

We now compute *p*(**u**|*y*) to predict utility estimates for all health states in the sample. This is obtained by integrating out equation (7) with respect to **α** and the hyperparameters γ, τ^2^, *v*^2^, β, and σ^2^. It follows from equation (7) that the posterior distribution of **θ** is not in the closed form. This implies that the Markov Chain Monte Carlo (MCMC) methods are then needed. Full conditional posterior distributions for all parameters **u**, α, γ, τ^2^, *v*^2^, β, *and σ*^2^ using MCMC methods are all set out in Kharroubi (25).

To this end, it is important to correct utility to the population mean. Note that the distribution of the individual respondent effect α_*j*_ in (2) is defined as independent log-normal. This implies that the utility function u(**x**) in model (1) represents the population median utility for a health state **x** and not the required population mean utility which, using model (1), is defined as


$$\begin{array}{l} \bar{u}\left(\text{x}\right)=1-E\left(\alpha \right)\left\{1-u\left(\text{x}\right)\right\} \end{array}$$

where *E*(****α**)** represents the expected value of **α** over the total population. When *E*(****α**)** = 1, then $\bar{u}\left(\text{x}\right)$ will be the same as *u*(**x**).

In the context when no covariates are used, the distribution (2) of the respondent effects becomes $\alpha_{j}\sim LN\left(0,\tau^{2}\right)$. This results in *E*(**α**)**=***exp*(τ^2^/2). However, when there are covariates, it follows from (2) that


$$\begin{array}{l} E\left(\alpha \right)=E\left\{E\left(\alpha |\text{t}\right)\right\}=E\left\{\exp\left({\text{t}}^{T}\gamma \right)\right\}\;\exp\left(\tau^{2}/2\right), \end{array}$$

all of which are obtained directly from the MCMC simulation. Therefore, the calculation of *E* (**α**) is straightforward

All theoretical and technical details of the non-parametric Bayesian model are reported in Kharroubi et al. (23, 25). Matlab source code for implementing the Bayesian approach is available online in the Supplementary Material. Note that the codes are not generic and need to be modified as per users' specific purposes.

## Results

### Irish with Polish Prior

The Bayesian model (1) was first implemented to predict an Irish value set, where the Polish results were employed as informative priors (which we will refer to as combined analysis from now on). The resultant utility estimates were then compared to those obtained from analyzing the Irish data alone (which from now on we will refer to as single analysis).

Here, the vector of individual-specific covariates is set as (Age, Age^2^, Sex). Also the roughness parameters *b*_*d*_ in formula (5) is set to be $b_{d}=2.5/{\left(l_{d}-1\right)}^{2}$, where *l*_*d*_ denotes the number of levels in dimension *d* (23). This value of *b*_*d*_ is chosen because exp$\left\{-{\left(l_{d}-1\right)}^{2}b_{d}\right\}\;$represents the correlation between the utility values for two health states differing only in that one is at level 1 and the other at level *l*_*d*_ in dimension *d* (23).

**Figure 1:**
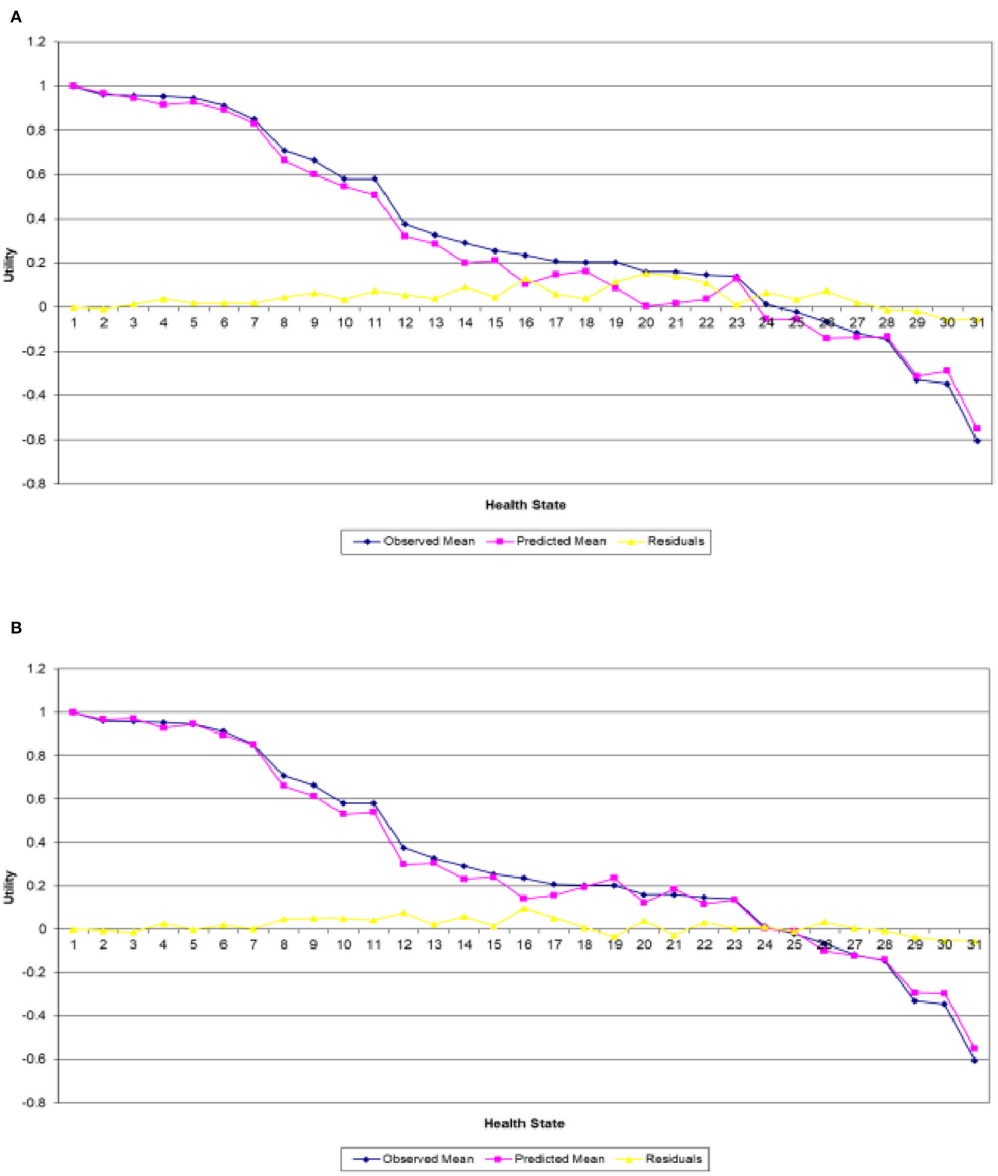
Actual and predicted mean health states valuations generated from analyzing. **(A)** Irish data only and **(B)** Irish data with Polish results as informative priors.

The MCMC sampler was allowed to iterate for 3,000 runs, with an initial run of 1,000 iterations as “burn-in” (these runs were discarded). Figures 1A,B presents the estimated (line in pink) and actual (line in blue) mean utilities for the 30 EQ-5D-5L health states valued in the Irish survey as well as the full health obtained from the combined and single analyses, respectively. The errors in both plots are displayed by the line in yellow and are obtained by calculating the difference between the two utilities. In both figures, the health states were ordered in terms of estimated mean utilities and were then plotted accordingly. The single analysis in Figure 1A exhibits a clear variation of the actual mean utilities around the estimated ones, particularly for the mild and worse health states, whereas Figure 1B clearly shows that the combined analysis predicts the mean utilities quite well across the full board.

**Figure 2:**
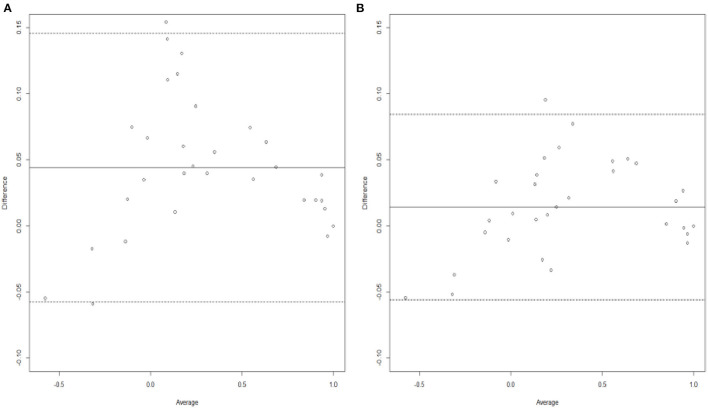
Bland-Altman plots generated from analyzing **(A)** Irish data only and **(B)** Irish data with Polish results as informative priors.

Another way to check the adequacy of the assumed models is to quantify the gains in terms of bias and/or precision. This is achieved by the Bland–Altman agreement plot that displays the difference values between predicted and actual mean utilities against the average bias (38). Figures 2A,B present the Bland–Altman agreement plot obtained from the combined and single analyses, respectively. The solid line in each plot represents the average bias, whereas the dotted lines are the 95% limits-of-agreement. It can be clearly seen that the combined analysis shows a much greater agreement than the single one. The rationale for this is based on the following three observations. Firstly, shorter width of the 95% limits of agreement, with values of 0.1402 for the combined analysis vs. 0.2030 for the single one. Secondly, smaller difference in average bias, with values of 0.0144 for the combined analysis and 0.0441. Thirdly, the standard deviation of the differences obtained from the combined analysis is also smaller than from the single analysis, with values of 0.0357 and 0.0518, respectively.

The overall impact of this can be seen from Table 1 which displays the inferences for the utilities of the 30 EQ-5D-3L states evaluated in the study as well as the perfect health. For each health state, Columns 2 and 3 present, respectively, the number of valuations together with the observed mean utility of the Irish data only, while Columns 4 and 5 show the Polish estimated mean utility and standard deviation that were used as informative priors in the combined analysis. Moreover, Columns 6 and 7 exhibit the predicted mean utility and standard deviation obtained from the single analysis, respectively, whereas Columns 8 and 9 show the corresponding estimates obtained from the combined analysis. As clearly seen throughout this comprehensive table, the combined analysis provides much better predictions compared to the single analysis overall, with RMSE of 0.038 vs. 0.068, respectively. Furthermore, it can also be seen that the posterior standard deviations of the utility estimates are larger for the single analysis. The posterior standard deviations for the combined analysis are smaller is due to the fact that it is a model that employs the Polish results as prior beliefs, hence producing better estimates.

**Table 1:** Estimates for utilities of the 30 EQ-5D-5L health states valued in the survey in addition to the full health.

**Health State**	**N**	**Observed Mean**	**Polish**		**Irish**		**Irish with Polish prior**	
			**Prior mean**	**Prior SD**	**Posterior mean**	**Posterior SD**	**Posterior mean**	**Posterior SD**
11111	0	1	1	0	1	0	1	0
11112	41	0.9122	0.9198	0.0351	0.8929	0.0364	0.8933	0.0318
11121	41	0.9549	0.9070	0.0399	0.9163	0.0430	0.9282	0.0374
11211	41	0.9622	0.9692	0.0388	0.9699	0.0397	0.9682	0.0341
11453	41	0.1598	0.1744	0.0672	0.0054	0.0641	0.1212	0.0602
12111	41	0.9463	0.9059	0.0365	0.9273	0.0363	0.9477	0.0324
12112	41	0.8500	0.8385	0.0384	0.8307	0.0381	0.8485	0.0344
13541	41	0.2024	0.2586	0.0627	0.1626	0.0584	0.1938	0.0525
14335	41	−0.0671	0.0655	0.0685	−0.1419	0.0658	−0.1005	0.0604
15224	41	0.2024	0.2639	0.0633	0.0874	0.0620	0.2358	0.0561
21111	41	0.9585	0.9280	0.0395	0.9456	0.0400	0.9714	0.0353
21514	41	0.2915	0.3510	0.0611	0.2007	0.0610	0.2320	0.0542
22245	41	−0.1171	0.1296	0.0652	−0.1373	0.0657	−0.1212	0.0591
23323	41	0.5805	0.6127	0.0498	0.5452	0.0496	0.5314	0.0431
24151	41	−0.0207	0.0919	0.0674	−0.0557	0.0640	−0.0103	0.0557
25432	41	0.2073	0.2582	0.0598	0.1470	0.0575	0.1558	0.0515
31125	41	0.2354	0.3022	0.0626	0.1050	0.0615	0.1399	0.0571
32533	41	0.3768	0.4407	0.0552	0.3208	0.0541	0.2997	0.0488
33252	76	0.1467	0.1508	0.0570	0.0362	0.0511	0.1151	0.0488
34444	41	−0.3463	−0.0071	0.0708	−0.2874	0.0710	−0.2946	0.0618
35311	41	0.6646	0.5602	0.0524	0.6011	0.0523	0.6139	0.0470
41231	41	0.7085	0.6712	0.0499	0.6639	0.0506	0.6612	0.0447
42354	41	−0.3293	−0.0225	0.0730	−0.3120	0.0717	−0.2924	0.0641
43415	41	0.0134	0.0797	0.0706	−0.0533	0.0644	0.0038	0.0589
44522	41	0.3268	0.3342	0.0597	0.2870	0.0539	0.3057	0.0491
45143	41	−0.1451	0.0978	0.0686	−0.1334	0.0677	−0.1400	0.0597
51342	41	0.1390	0.3050	0.0577	0.1284	0.0581	0.1344	0.0511
52421	41	0.5805	0.4888	0.0556	0.5060	0.0522	0.5390	0.0483
53134	41	0.1598	0.1768	0.0663	0.0184	0.0625	0.1854	0.0590
54213	41	0.2549	0.2913	0.0594	0.2097	0.0559	0.2407	0.0507
55555	123	−0.6053	−0.2506	0.0737	−0.5505	0.0679	−0.5509	0.0638
**RMSE**					0.068		0.038	

*UK: United Kingdom; SD: standard deviation; RMSE: Root Mean Square Error*.

### Polish with Irish Prior

We now apply model (1) to predict a Polish value set, where the Irish results were now employed as informative priors (combined analysis), and the resultant utility estimates were then compared to those obtained from analyzing the Polish data alone (single analysis).

**Figure 3:**
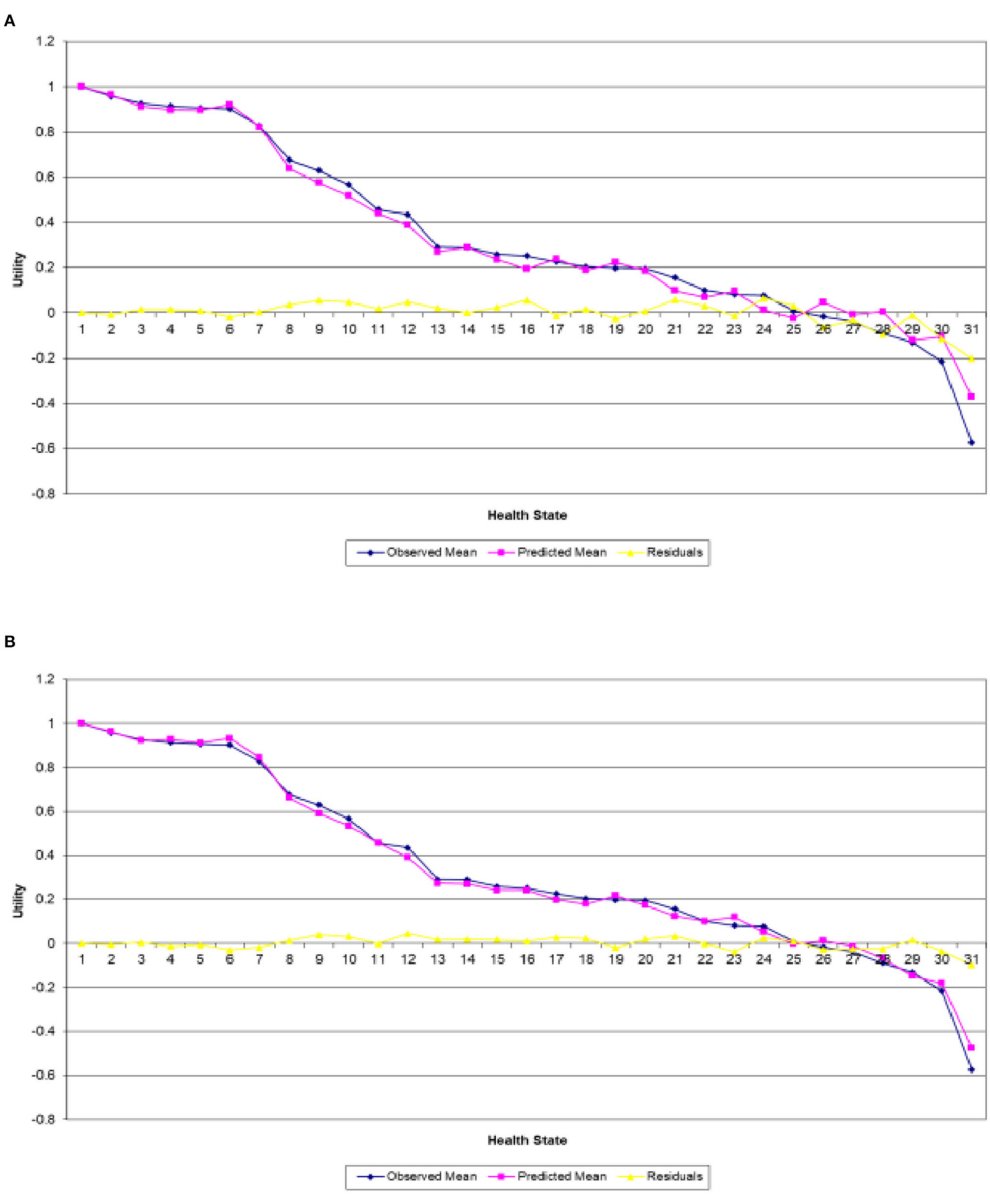
Actual and predicted mean health states valuations generated from analyzing **(A)** Polish data only and **(B)** Polish data with Irish results as informative priors.

In a similar way to section Irish With Polish Prior, Figures 3A,B present the predicted and actual mean utility estimates for the 30 EQ-5D-5L health states valued in the Polish survey along with their differences obtained from the combined and single analyses, respectively. As is the case in section Irish With Polish Prior, Figure 3A exhibits a clear variability of the actual values around the estimated mean utilities, particularly for the mild and worse health states, while Figure 3B clearly shows that that the combined analysis predicts the mean utilities quite well for almost all heath states in the study.

**Figure 4:**
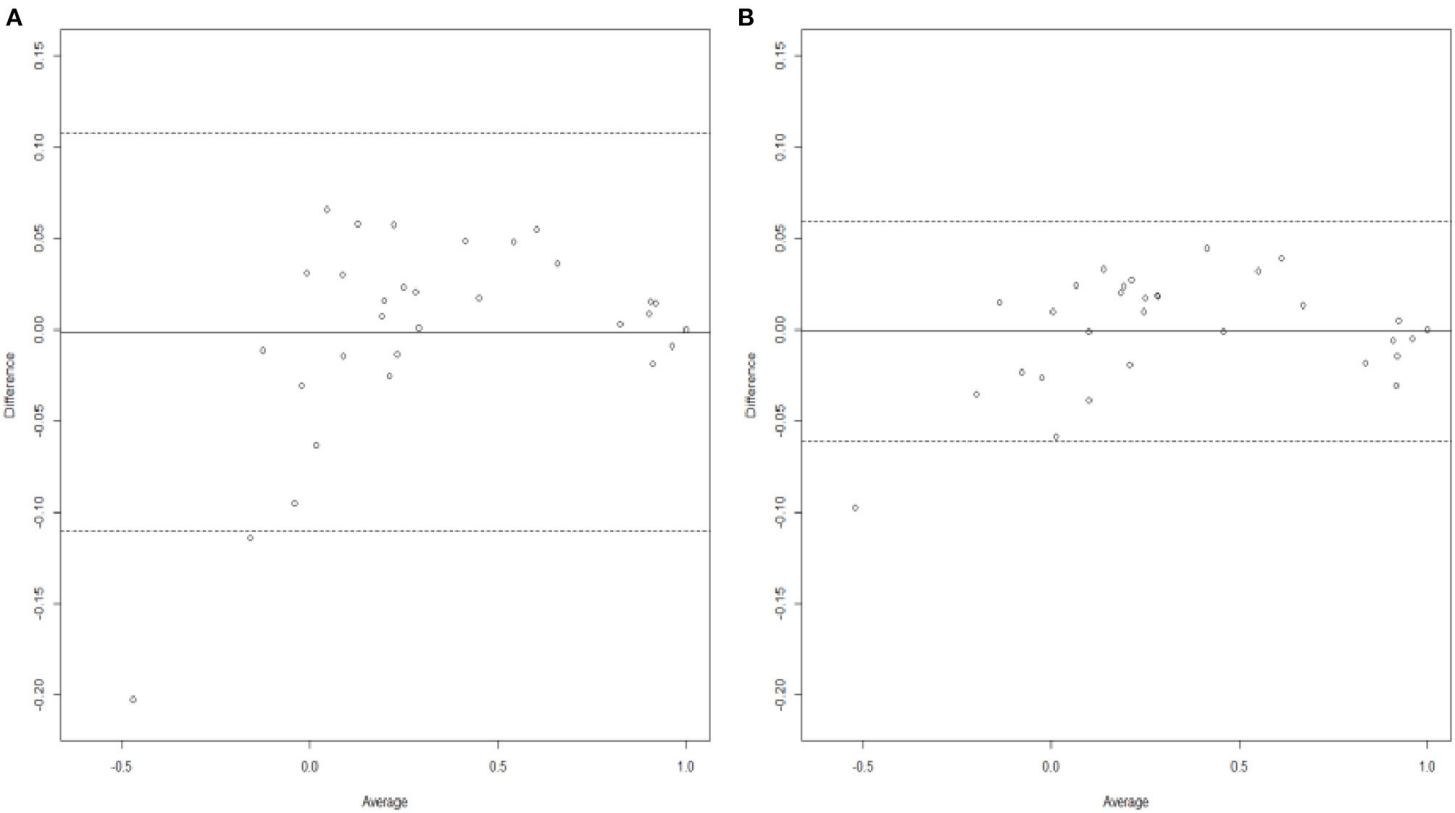
Bland-Altman plots generated from analyzing **(A)** Polish data only and **(B)** Polish data with Irish results as informative priors.

When comparing the two analyses using the Bland–Altman agreement plots (Figures 4A,B), we can also see clearly that the combined analysis has performed better in terms of a greater agreement. As in section Irish With Polish Prior, this is also due to (1) shorter width of the 95% limits of agreement, with values of 0.1205 for the combined analysis compared to a value of 0.2186 for the single one, (2) smaller difference in mean bias, with values of −0.0006 for the combined analysis and −0.0011, and (3) smaller standard deviation of the differences, with values of 0.0307 for the combined analysis vs. 0.0557 for the single analysis. Finally, and in a similar pattern to Table 1, it can be clearly seen throughout the comprehensive Table 2 that the combined analysis provides much better predictive performance when compared to the single analysis overall, with a value of 0.030 for RMSE against 0.055 from the single analysis. The posterior standard deviations of the utility estimates are also smaller for the combined analysis.

**Table 2:** Estimates for utilities of the 30 EQ-5D-5L health states valued in the survey in addition to the full health.

**Health State**	***N***	**Observed Mean**	**Irish**		**Polish**		**Polish with Irish prior**	
			**Prior mean**	**Prior SD**	**Posterior mean**	**Posterior SD**	**Posterior mean**	**Posterior SD**
11111	0	1	1	0	1	0	1	0
11112	39	0.9269	0.9044	0.0326	0.9122	0.0385	0.9218	0.0322
11121	40	0.9137	0.9357	0.0385	0.8981	0.0437	0.9278	0.0362
11211	39	0.9577	0.9715	0.0355	0.9663	0.0425	0.9624	0.0349
11453	40	0.0812	0.1503	0.0574	0.0956	0.0736	0.1196	0.0622
12111	40	0.9062	0.9531	0.0325	0.8969	0.0400	0.9122	0.0325
12112	40	0.8262	0.8643	0.0341	0.8230	0.0420	0.8444	0.0348
13541	40	0.2037	0.2780	0.0523	0.1878	0.0687	0.1796	0.0579
14335	40	0.0075	0.0144	0.0589	−0.0238	0.0751	−0.0025	0.0637
15224	40	0.2513	0.1902	0.0555	0.1937	0.0693	0.2412	0.0591
21111	40	0.9025	0.9744	0.0359	0.9211	0.0433	0.9328	0.0352
21514	40	0.2900	0.3122	0.0546	0.2890	0.0669	0.2711	0.0561
22245	39	−0.0167	−0.0041	0.0588	0.0465	0.0714	0.0417	0.0628
23323	39	0.6308	0.5803	0.0444	0.5757	0.0546	0.5914	0.0467
24151	39	−0.0897	0.0952	0.0573	0.0052	0.0738	−0.0664	0.0631
25432	39	0.1949	0.2439	0.0515	0.1873	0.0655	0.1743	0.0577
31125	40	0.2587	0.2297	0.0550	0.2355	0.0686	0.2414	0.0584
32533	39	0.4359	0.3728	0.0484	0.3873	0.0605	0.3911	0.0523
33252	79	0.1000	0.1716	0.0458	0.0697	0.0625	0.1006	0.0541
34444	39	−0.2167	−0.1415	0.0636	−0.1032	0.0775	−0.1815	0.0677
35311	40	0.5662	0.6542	0.0468	0.5182	0.0574	0.5341	0.0503
41231	40	0.6763	0.6966	0.0453	0.6398	0.0546	0.6628	0.0458
42354	40	−0.1313	−0.1575	0.0642	−0.1201	0.0800	−0.1462	0.0685
43415	40	−0.0387	0.1078	0.0576	−0.0081	0.0773	−0.0126	0.0642
44522	40	0.2913	0.3782	0.0483	0.2706	0.0654	0.2729	0.0547
45143	40	0.0775	−0.0210	0.0606	0.0116	0.0752	0.0529	0.0663
51342	39	0.2256	0.2248	0.0521	0.2386	0.0632	0.1983	0.0564
52421	40	0.4575	0.5871	0.0467	0.4400	0.0609	0.4583	0.0507
53134	40	0.1562	0.1451	0.0560	0.0982	0.0727	0.1230	0.0616
54213	39	0.1987	0.3200	0.0501	0.2236	0.0651	0.2179	0.0557
55555	119	−0.5723	−0.3710	0.0608	−0.3701	0.0808	−0.4750	0.0718
**RMSE**					0.055		0.030	

*UK, United Kingdom; SD, standard deviation; RMSE, Root Mean Square Error*.

## Discussion

Kharroubi (25) built a non-parametric Bayesian model that allows the already existing results from one country to be employed as a potential prior information in another country. Here, we explored the use of this method for Polish migrants and native Irish data modeling Irish (Polish) data alongside small Polish (Irish) samples to generate Polish (Irish) estimates. The resultant new estimates were then compared to those obtained modeling the data from each country alone. The findings proved that existing countries' valuations could be used as informative priors to produce better utility estimates under all criterions used, including estimated against actual mean utilities, mean predicted error and RMSE. This sort of analysis (adopting strength from existing countries) will be greatly important in countries where large-scale evaluation exercises are hard to conduct, especially for countries with small population size or LMICs.

Despite the present paper not offering new methodological advances, the novelty here was to investigate the use of non-parametric Bayesian model for countries with smaller design valuation studies and different population compositions, type of work, cultures and languages. All of these could have an impact on the relative valuations of the dimensions of health (such as, self-care and anxiety/depression) and on where-about each health state lies on the [0–1] dead-perfect health scale. This suggests that the approach presented here may not generate precise utility estimates all the time. Further, the analysis presented here also provided a re-assuring story regarding the superiority and flexibility of the non-parametric Bayesian approach in using existing preference data, thereby generating accurate utility estimates.

It is true from the two analysis presented here that the improvement in the utility estimates in general and in the mean-squared error in particular is moderate. However, there are other crucial benefits especially those related to health and quality of life gains. As lots of reimbursement agencies worldwide need cost-effectiveness assessments, effectiveness analysis would become more international with combination of data across different countries. The valuation of health states for calculating QALYs would be a key component of this process which, in turn, requires accurate utility estimates. For example, results from Table 1 revealed that the combined analysis provides much better predictive performance when compared to the single analysis overall, with values for RMSE of 0.038 and 0.068, respectively. Therefore, the difference in utility estimates is, on average, equal to 0.03, which leads to an increase in QALYs from 0.5 to 0.53 for a treatment that extends life by an extra year. This in turn leads to a decrease in the cost per QALY from £20,000 to £18, 867 for a treatment costing £10,000, and so puts it under the cost-effectiveness threshold employed by the National Institute for Health and Clinical Excellence (NICE) in the UK. As a result, this could potentially influence the probability of whether a new treatment or health care scheme is deemed cost-effective and funded. It could also impact on the validity of the resource allocation decisions being made. Heijink et al. (39) drew similar conclusion from their analysis on the impact of different valuation functions on QALYs.

A key benefit of the non-parametric Bayesian model presented here is that it allows for multi-countries to be analyzed rather than two. Further, equations (3) and (4) may be generalized further to handle *n* (*n* > 2) countries. Thus, we formally assign a multivariate normal distribution for *u*(**x**) with mean:


(7) $$\begin{array}{l} E\left(u\left(\text{x}\right)\right)=\sum_{k=1}^{n}E\left(u_{k}\left(\text{x}\right)\right)+\gamma +\beta^{T}\text{x} \end{array}$$

and variance–covariance matrix


(8) $$\begin{array}{l} \text{cov }\left(u\left(\text{x}\right),u\left({\text{x}}^{\prime }\right)|\sigma^{2}\right)=\sum_{k=1}^{n}\text{cov}\left(u_{k}\left(\text{x}\right),u_{k}\left({\text{x}}^{\prime }\right)\right)+\sigma^{2}c\left(\text{x},{\text{x}}^{\prime }\right) \end{array}$$

where $\overset{n}{\underset{k=1}{\sum }}E\left(u_{k}\left(\text{x}\right)\right)$ is the overall expected utility of state **x** and $\overset{n}{\underset{k=1}{\sum }}cov\left(u_{k}\left(\text{x}\right),u_{k}\left({\text{x}}^{\prime }\right)\right)\;$is the overall variance–covariance matrix between the two utility functions *u*_*k*_(**x**) and $u_{k}\left({\text{x}}^{\prime }\right)\neg \neg$ for two distinct health states **x** and **x'**, both of which are computed directly from modeling the existing datasets in *n* different countries. Work is in progress on demonstrating this idea for SF-6D in the UK, Hong Kong and Japan has introductory results that are particularly promising.

As already mentioned, generic measures of HRQoL, such as the EQ-5D and SF-6D, have been valued in different countries and so there are many different value sets from different countries and subgroups available. Such valuation studies are very expensive and are potentially wasteful. The analysis presented here demonstrates how existing countries' valuations could be used as informative priors to produce better utility estimates. This offers the potential to reduce the need for conducting large surveys in every country which in turn will reduce the cost of cross-country valuation. The approach presented here (borrowing strength from existing countries) could be particularly promising for countries where large-scale evaluation exercises are hard to conduct, especially for countries with small population size or LMICs. For instance, it is worth noting that large-scale national EQ-5D-5L valuations studies are significant undertakings of research that require substantial resources and logistics to be completed. These studies require a minimum of 1,000 respondents to value 86 health states, as mentioned above. As such, using the methods employed in this paper and a similar smaller design EQ-5D-5L valuation study to Kelleher et al. (33), future national valuation studies could be conducted more efficiently by having less respondents to value less health states compared to current large-scale national valuation studies. Examining this could be particularly promising and would form a key research agenda for further research. In addition, further analysis could be conducted more efficiently using simulated data. The thing is that, if through simulated data we know how the value sets differ, then we can explore the relationship between how different the countries are and how useful the use of priors are. However, although the empirical example is helpful and a worthwhile addition to the literature in its own right, it does not allow exploration of the full range of distances between national value sets (24). Further research is encouraged to examine this.

The present study has certain limitations that ought to be considered. First, the study sample size was small which may in some sense limit the generalizability of the utility values obtained. Second, snowball sampling has been used, thus a good representative sample of Polish migrants and/or native Irish was not selected, let alone there was some difficulties associated with obtaining a good representative sample [see Kelleher et al. (33) for an overview]. This implies that the results of this study ought not to be considered as good representative of Polish migrants and/or native Irish in Ireland. Third, the small number of health states (i.e., 30 EQ-5D-5L health states) valued in this study could have an impact on the precision of econometric modeling, suggesting that the presented EQ-5D-5L utility estimates may not be considered as representative of the general Polish migrants or native Irish population. Future research with more representative samples is then encouraged to produce the Polish migrants or native Irish specific EQ-5D-5L value set. However, because of the way the non-parametric Bayesian model is defined, this should not in theory impact on the resulting utility estimates from this paper, though this could be further examined in future work.

In conclusion, the promising results suggest that existing countries' valuations could be used as informative priors to generate better utility estimates than modeling the data from each country separately. This kind of analysis could be particularly promising in terms of reducing the need for conducting large surveys in every country which in turn would reduce the cost of cross-country valuation. This will be greatly important for countries where large-scale evaluation exercises are expensive and hard to conduct. Similar approach could be used to other descriptive measures like HUI-II (40), in addition to other condition-specific measures (41). Ongoing research is underway to examine this

## Data Availability Statement

The raw data supporting the conclusions of this article will be made available by the authors, without undue reservation.

## Ethics Statement

The studies involving human participants were reviewed and approved by NUI Galway's Research Ethics Committee (application number 18-Mar-13). The patients/participants provided their written informed consent to participate in this study.

## Author Contributions

SK participated in the conceptualization of the idea, the design of the methodology, software, data analysis, data interpretation, manuscript drafting, and the final review of the manuscript. DK reviewed and finalized the manuscript and participated in the conceptualization of the study. All authors have read and approved the final manuscript.

## Funding

This research was funded by the University Research Board (URB) at the American University of Beirut (AUB) awarded to SK (Grant No. 104110). The original study was funded by the Hardiman Research Scholarship 2017 awarded by NUI Galway to DK.

## Conflict of Interest

The authors declare that the research was conducted in the absence of any commercial or financial relationships that could be construed as a potential conflict of interest.

## Publisher's Note

All claims expressed in this article are solely those of the authors and do not necessarily represent those of their affiliated organizations, or those of the publisher, the editors and the reviewers. Any product that may be evaluated in this article, or claim that may be made by its manufacturer, is not guaranteed or endorsed by the publisher.
